# Temporary tracheotomy for post-intubation laryngeal edema after lung cancer surgery: a case report

**DOI:** 10.1186/s13019-023-02187-4

**Published:** 2023-03-20

**Authors:** Yoshihito Iijima, Yuki Takaoka, Nozomu Motono, Hidetaka Uramoto

**Affiliations:** 1 Department of Thoracic Surgery, Kanazawa Medical University, 1-1 Daigaku, Uchinada-Machi, Kahoku-gun, Ishikawa 920-0293 Japan; 2 Department of Head and Neck Surgery, Kanazawa Medical University, 1-1 Daigaku, Uchinada-machi, Kahoku-gun, Ishikawa 920-0293, Japan

**Keywords:** Double-lumen tube, Lung cancer, Post-intubation laryngeal edema, Temporary tracheostomy, Thoracic surgery

## Abstract

**Background:**

In the post-intubation period, laryngeal edema is one of the most severe complications, which can cause significant morbidity and even death. Herein, we report a case in which we performed a temporary tracheostomy during surgery because of the risk of postoperative laryngeal edema, successfully avoiding post-intubation laryngeal edema complications.

**Case presentation:**

A 78-year-old man underwent surgery for left upper lobe lung cancer. He had a history of chemoradiotherapy for laryngeal cancer, bronchial asthma, and chronic obstructive pulmonary disease. He was diagnosed with grade 1 laryngeal edema using computed tomography, and there was a risk of developing post-intubation laryngeal edema. Additionally, there was a decrease in laryngeal and pulmonary functions; therefore, postoperative aspiration pneumonia was judged to be a fatal risk. A temporary tracheostomy was performed during surgery to avoid postoperative intubation laryngeal edema. He was found to have exacerbated laryngeal edema, which is a serious complication of airway stenosis.

**Conclusions:**

Temporary tracheostomy should be considered to avoid airway stenosis due to post-intubation laryngeal edema in patients with laryngeal edema after radiotherapy.

## Background

Laryngeal edema commonly occurs after tracheal intubation [1]. Post-intubation laryngeal edema (PILE) is a severe complication, that causes significant morbidity and death [1, 2]. Urgent treatment, such as tracheostomy may be required depending on the degree of stenosis. In thoracic surgery, a double-lumen tube (DLT) is used for differential lung ventilation, but the DLT is thicker than a single lumen tube, and there are reports of PILE or airway stenosis [2–6]. We report a case where PILE complications were successfully avoided by performing a temporary tracheostomy (TT) during surgery in a patient with a high risk of developing postoperative edema.

## Case presentation

**Fig. 1:**
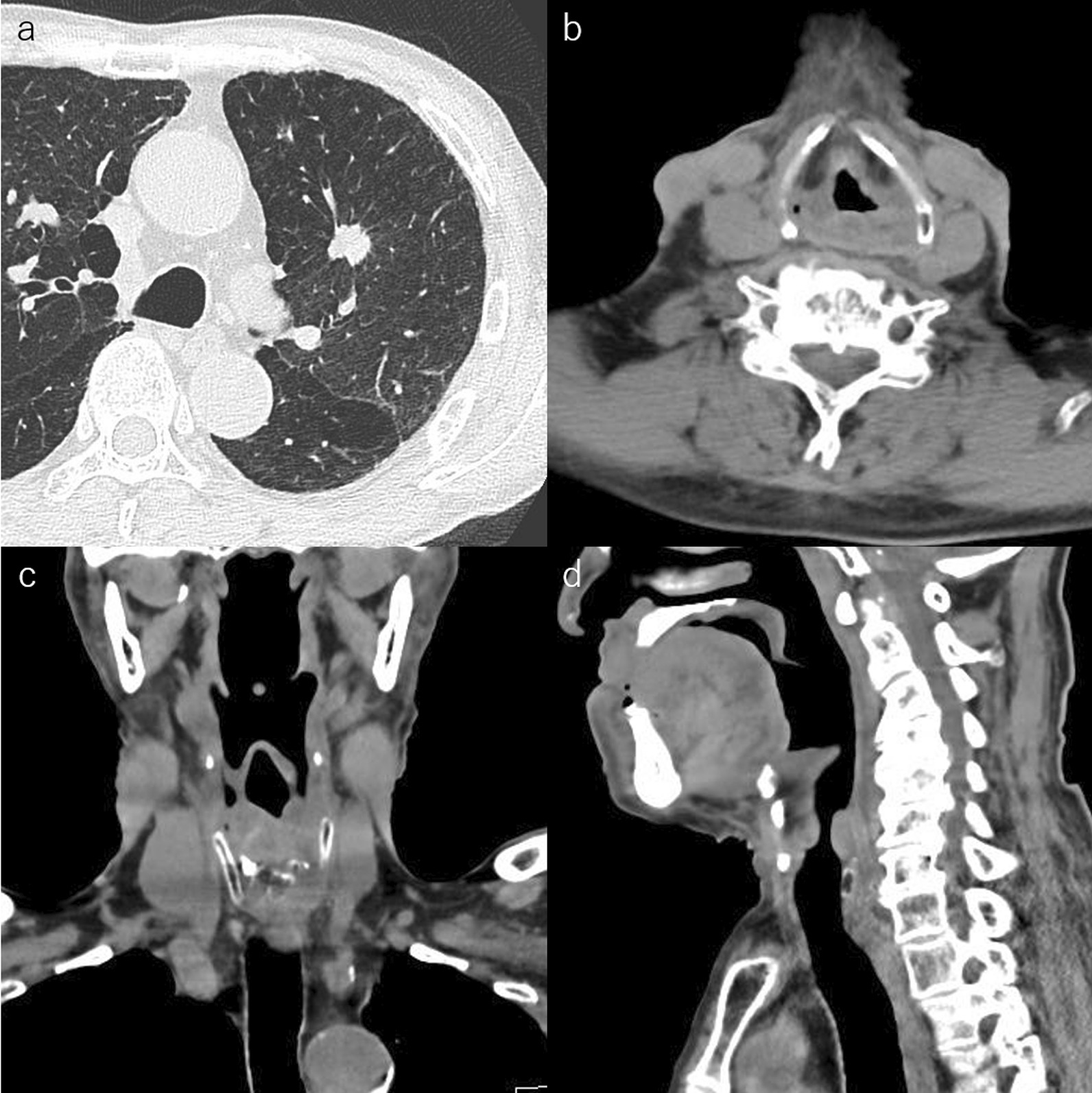
Computed tomography images. Computed tomography showing **a** a solid nodule shadow in left ventral segment (S3), and laryngeal edema. **b** Horizontal view, **c** coronal view, and **d** Sagittal view

**Fig. 2:**
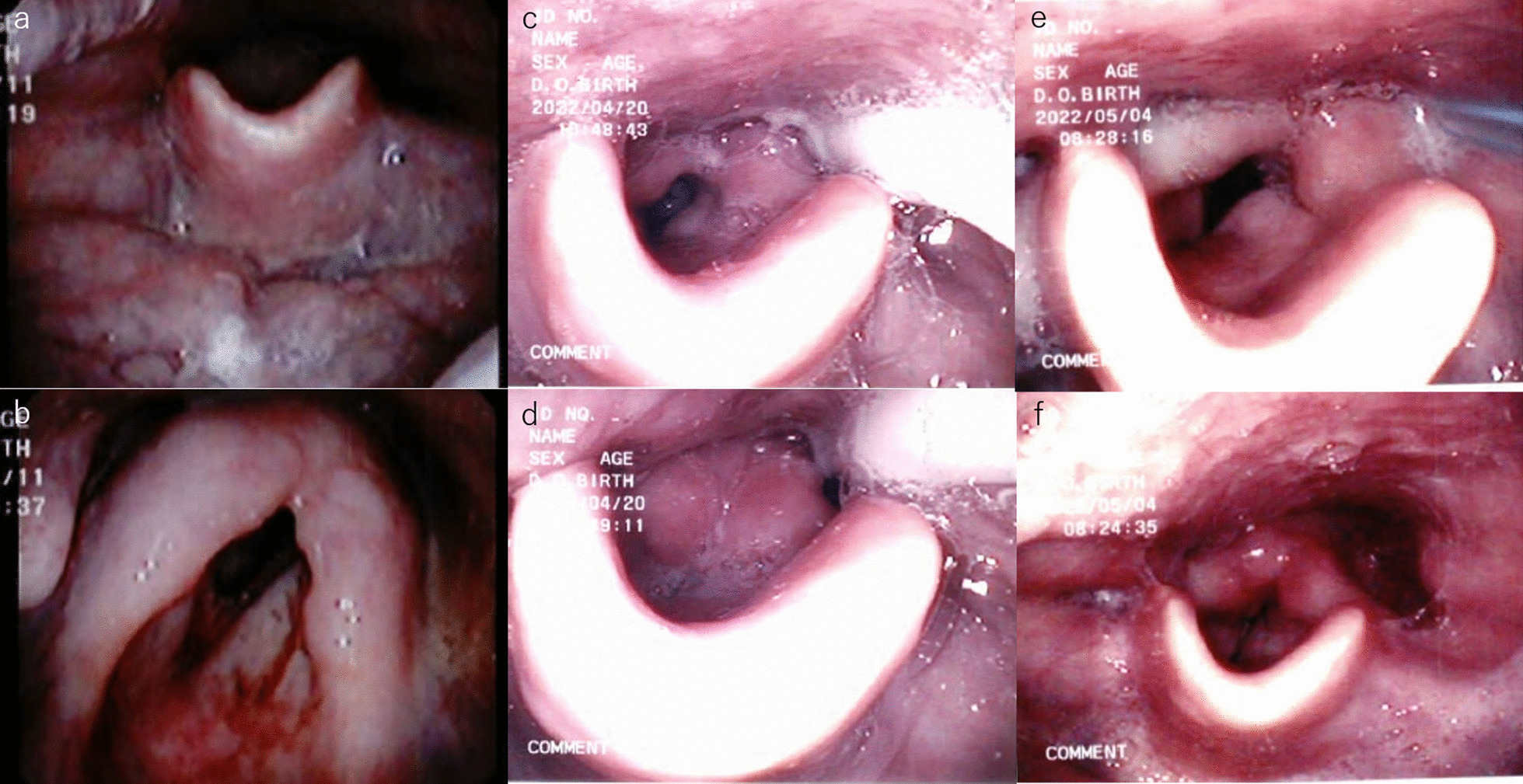
Perioperative laryngoscope findings. **a**, **b** Preoperative. Radiation Therapy Oncology Group (RTOG) grade 1 laryngeal edema was observed. **c**, **d** Postoperative day 2. RTOG grade 3 laryngeal edema was observed. **e**, **f** Postoperative day 16. RTOG grade 2 laryngeal edema was observed

A 78-year-old man was found to have a nodule in the upper lobe of the left lung. His height and weight were 161 cm and 57 kg, respectively. The patient had been treated 3 years and 6 months earlier with three courses of chemotherapy with 80 mg/m^2^ cisplatin and a total of 70 Gy of radiotherapy for stage III laryngeal cancer. Additionally, he had a history of bronchial asthma and chronic obstructive pulmonary disease. Squamous cell carcinoma was diagnosed by transbronchial lung biopsy and staged as cT1bN0M0, stage IA2 lung cancer. Therefore, the patient was referred for surgery. A chest computed tomography (CT) scan showed a solid nodule shadow in the left ventral segment measuring 1.5 × 1.4 cm (Fig. 1a) without any lymphadenopathy. CT findings showed edema around the arytenoid and vocal cords (Fig. 1b–d) and the inner tracheal diameter at the cricoid level was 11.5 × 6.5 mm. Interestingly, 2-deoxy-2-(18F)-fluorodeoxyglucose positron emission tomography revealed tracer accumulation in the lung nodule. There was no accumulation suggesting local recurrence of laryngeal cancer. Laryngoscopy showed swelling of both vestibular folds, whitening of the mucosa, and edema from the oropharynx to the hypopharynx [grade 1 laryngeal edema in the radiation therapy oncology group (RTOG)]. The vocal cords had good mobility, but poor laryngeal elevation and mild saliva retention with diminished laryngeal perception were observed (Fig. 2a, b). The head and neck surgeon determined that there was a risk of postoperative exacerbation of laryngopharyngeal edema due to intubation. The forced expiratory volume in one second (FEV1) was 1050 mL, %FEV1 was 42.6%, and FEV1/forced vital capacity (FEV1%) was 34.3% showing a remarkable decrease. There was a decrease in laryngeal and pulmonary functions; therefore, postoperative aspiration pneumonia was judged to be a fatal risk. At a conference including a thoracic surgeon, head and neck surgeon, and anesthetist, it was determined that the patient was at risk of potentially fatal postoperative aspiration pneumonia and laryngeal edema which could be exacerbated postoperatively. Accordingly, TT was performed during surgery.

To prevent bronchospasm, 30 mg of prednisolone was infused preoperatively. The anesthesiologist confirmed the space in the subglottic space with laryngoscopy and determined that DLT intubation was possible. The 35-French DLT was inserted without resistance. The cuff-leak test was negative. Left upper division segmentectomy and nodal dissection 1b were performed in the right lateral position. Then, a tracheostomy was performed in the supine position. The durations of the lung surgery, tracheostomy, and anesthesia were 137, 22, and 239 min, respectively. After tracheostomy, the larynx examination revealed that the laryngeal edema was worse than before intubation.

On postoperative day (POD) 2, laryngoscopy showed swelling and stenosis of the glottis from the arytenoid muscle, and moderate saliva retention and aspiration were observed (RTOG grade 3) (Fig. 2c, d). A videoendoscopic evaluation of swallowing performed on POD 4 revealed a decrease in pharyngeal contractility; therefore, a swallowing training diet was initiated. The chest drainage tube was removed on POD 7 because of prolonged air leakage. We determined that postoperative swallowing function training and respiratory rehabilitation were necessary and transferred to the Head and Neck Surgery Department to continue rehabilitation. The swelling of the larynx was gradually improved on POD 16 (RTOG grade 2) (Fig. 2e, f). The tracheostomy was closed on POD 28 and the patient was discharged on POD 30.

## Discussion and conclusion

Traumatic laryngitis is a complication associated with DLT placement [7]. PILE results from trauma to the laryngeal mucosa causing mucosal edema and swelling. Approximately 54% of the patients who have undergone intubation or tracheostomy have subvocal ulcers, and 93% have mucosal inflammation or edema [1]. Therefore, the DLT should be of appropriate size and according to the patient’s sex, physique, and imaging data (CT or chest radiography) [7, 8].

Female individuals have anatomically smaller airways than male individuals and, therefore, they are at a higher risk of developing PILE [7, 9]. To our knowledge, there are 11 reported cases of PILE after lung surgery using DLT (including ours), in the English and Japanese literature (Table 1) [2–6]. Of these, 10 were of female patients and seven cases with ≤ 150 cm height. There was resistance during intubation in eight cases, and in four cases, the DLT size was reduced. If there are risk factors, such as female sex or short stature, the preparation of a smaller size of DLT or single lumen intubation tube with bronchial blocker is necessary. Moreover, if there is resistance during intubation, it may be important to select a smaller size intubation tube or perform TT.

**Table 1:** Summary of post-intubation laryngeal edema after lung surgery cases reported in the litarature

Case	Age (years)	Sex	Hight (cm)	Weight (kg)	Head and neck cancer	Asthma	Cormack Lehane Grade	Inner diameter of the narrowest part (mm)	DLT size (Fr)	Resistance during intubation	Operation	Operation time (min)	Anesthesia time (min)	Diagnosis date (POD)	Steroid	Airway management	Date of tracheostomy (POD)	References
1	76	F	155.8	52.1	–	+		ND	35	+	LUL + ND2a-1	95	140	1	mPSL	Emergency tracheostomy	1	[3, 4]
2	81	F	148	46	–	−	ND	ND	35	+	WR	59	95	3	DEX	Reintubation ↓ tracheostomy	8	[3, 4]
3	73	F	146	42	–	−	ND	ND	35 → 32 → 28	+	LS6 Seg	ND	ND	2	mPSL	Cricothyroid incision	2	[3, 4]
4	83	F	149.3	57.5	–	−	I	slightly stenosis	35 → 32	+	RUL + WR	267	ND	On the day of surgery	mPSL	Reintubation ↓ tracheostomy	3	[3, 4]
5	81	F	142	47	–	−	ND	ND	35	+	WR	ND	134	1	DEX	Emergency tracheostomy	3	[3, 4]
6	81	F	146.1	51.3	–	−	ND	10.4	35 → 32	+	LUL + ND2a-1	290	341	2	HCSS	Emergency tracheostomy	2	[3, 4]
7	80	F	140	41	–	−	I	13.9 × 11.4	32 → 28	+	LUL	ND	265	2	HCSS	Emergency tracheostomy	2	[4]
8	83	F	144.1	48.7	–	−	ND	ND	35	+	LUL + ND2a-1	204	280	3	mPSL	Reintubation ↓ tracheostomy	6	[5]
9	71	F	ND	ND	–	−	ND	ND	37	–	left pleurodesis	ND	ND	2	ND	Reintubation ↓ tracheostomy	4	[6]
10	49	F	163	64	–	−(atopy)	ND	ND	37	–	lung tumor biopsy	ND	ND	On the day of surgery	HCSS	Difficulty extubation (extubation after 48 h)	–	[2]
11	78	M	161	57	+	+	II	11.5 × 6.5	35	–	LUD Seg + ND1b	176	239	On the day of surgery	PSL	Scheduled tracheostomy	On the day	Our case

*DLT* double lumen tube, *POD* post operative day, *ND* not discribed, *LUL* left upper lobectomy, *ND* nodal dissection, *WR* wedge resection, *LS6 Seg* left S6 segmentectomy, *RUL* right upper lobectomy, *LUD seg* left upper division segmentectomy, *mPSL* methyl-predonisolone, *DEX* dexamethasone, *HCSS* hydrocortisone sodium succinate, *PSL* predonisolone

Our patient was male with an adequate to small size DLT [7] and smooth intubation. There were no previously reported risk factors in such cases; however, this patient had previously been treated with chemoradiotherapy (CRT) and larynx edema was noted preoperatively. Although radiotherapy (RT) allows laryngeal preservation in patients with head and neck cancers, it may induce salivary or laryngopharyngeal dysfunction. Fibrotic changes following RT may lead to lymphatic vessel obstruction, causing edema, particularly in the supraglottic areas [10]. Laryngeal edema after radiotherapy is strictly correlated with various dosimetric parameters, such as the mean dose [11] and the equivalent uniform dose [12]. There were three case reports of PILE in patients with a history of neck surgery or radiotherapy (Table 2) [13, 14]. Moreover, there is a previous report of onset 10 years after cervical treatment [13]. This suggests that previous cervical treatment may be a risk factor for developing PILE at any time. Additionally, the patient had a history of asthma and atopy, which can predispose him to an increased inflammatory response, resulting in severe edema and swelling [2]. Comorbidity of allergic disease and a history of CRT or neck surgery for laryngeal cancer or thyroid disease may be risk factors for PILE. Furthermore, decreased laryngeal function is a risk factor for severe aspiration pneumonia. In this case, decreased FEV1, %FEV1, and FEV1% were observed, and postoperative aspiration pneumonia was considered extremely fatal. Therefore, we recommend that TT may be considered to avoid airway stenosis due to PILE and postoperative aspiration pneumonia if there is pre-existing edema, a predisposition to allergies, and a decrease in laryngeal and pulmonary functions. However, TT for PILE is controversial. The postoperative observations could have been handled by performing a tracheotomy when laryngeal edema and laryngeal stenosis were present. There is also a risk of tracheal stenosis after tracheostomy. It is important to perform TT in appropriate cases, and accumulate future cases.

**Table 2:** Summary of post-intubation laryngeal edema after non-head and neck surgery cases having a history of head and neck surgery or radiotherapy

Case	Age (years)	Sex	Hight (cm)	Weight (kg)	Prior therapy for head and neck cancer					Primary disease	Operation	Tube type (size)	Resistance during intubation	Operation time (min)	Anesthesia time (min)	Diagnosis date (POD)	Steroid	Airway management	References
					Primary cancer	Surgery	Neck dissection	Radiotherapy	Interval (years)										
1	76	F	155.8	52.1	Tougue cancer	Excision of tougue base	Bilateral	+	10	Metastatic hepatic tumor	Segmental liver resection, cholecystectomy, rediofrequency ablation	SLT (ID 7.0 mm)	–	240	ND	On the day of surgery	DEX	Emergency tracheostomy	[13]
2	69	F	150	43	Thyroid cancer	Total thyroidectomy	Bilateral	−	7	Bilateral ureter stones	Transurethral ureterolithotripsy	SLT (ID 7.5 mm)	–	45	ND	On the day of surgery	DEX	Reintubation ↓ tracheostomy	[14]
3	78	M	161	57	Laryngeal cancer	–	–	+	3.5	Left lung cancer	Left upper division segmentectomy	DLT (35 Fr)	–	176	239	On the day of surgery	PSL	Temporary tracheostomy	Our case

*POD* post operative day, *SLT* single lumen tube, *DLT* double lumen tube, *ID* inner diameter, *ND* not discribed, *DEX* dexamethasone, *PSL* predonisolone

In conclusion, TT may be an important option for postoperative airway management in patients with laryngeal edema, airway stenosis, and decreased laryngeal function due to treatment for laryngeal cancer.

## Data Availability

All data generated or analyzed during this study are included in this published article.

## Abbreviations

CT: Computed tomography
DLT: Double-lumen tube
FEV1: Forced expiratory volume in one second
FEV1%: FEV1/forced vital capacity
PILE: Post-intubation laryngeal edema
POD: Postoperative day
CRT: Chemoradiotherapy
RT: Radiotherapy
RTOG: Radiation therapy oncology group
TT: Temporary tracheostomy
